# Brain Region Specific Pre-Synaptic and Post-Synaptic Degeneration Are Early Components of Neuropathology in Prion Disease

**DOI:** 10.1371/journal.pone.0055004

**Published:** 2013-01-30

**Authors:** Zuzana Šišková, Richard A. Reynolds, Vincent O’Connor, V. Hugh Perry

**Affiliations:** 1 Centre for Biological Sciences, University of Southampton, Southampton, United Kingdom; 2 Deutsches Zentrum für Neurodegenerative Erkrankungen, University of Bonn, Bonn, Germany; University of Leicester, United Kingdom

## Abstract

Synaptic abnormalities, one of the key features of prion disease pathogenesis, gives rise to functional deficits and contributes to the devastating clinical outcome. The synaptic compartment is the first to succumb in several neurodegenerative diseases linked with protein misfolding but the mechanisms underpinning this are poorly defined. In our current study we document that a focal intrahippocampal injection of the mouse-adapted 22L scrapie strain produces a complex, region-specific pathology in the brain. Our findings reveal that early synaptic changes in the stratum radiatum of the hippocampus, identical to those observed with the ME7 strain, occur when 22L strain is introduced into the hippocampus. The pathology was defined by degenerating Type I pre-synaptic elements progressively enveloped by the post-synaptic density of the dendritic spine. In contrast, the pathology in the cerebellum suggested that dendritic disintegration rather than pre-synaptic abnormalities dominate the early degenerative changes associated with the Purkinje cells. Indeed, both of the major synaptic inputs into the cerebellum, which arise from the parallel and climbing fibers, remained intact even at late stage disease. Immunolabeling with pathway selective antibodies reinforced these findings. These observations demonstrate that neuronal vulnerability to pathological protein misfolding is strongly dependent on the structure and function of the target neurons.

## Introduction

Prion diseases or transmissible spongiform encephalopathies (TSEs) are a family of progressive, invariably fatal, neurodegenerative diseases, affecting both animals and humans. The common underlying feature associated with development of the neuropathology is the presence of an abnormally folded, protease resistant (PrP^Sc^) form of the endogenous cellular prion protein (PrP^C^) in the brain and in the lymphoid tissues [1]. Scrapie, a naturally occurring prion disease of sheep and goats, has been widely studied in mouse models. Different murine prion strains are known to exhibit characteristic neuropathologies, which differ in the rate of disease progression, the extent of brain vacuolation and region-specific neuronal loss [2]. It is thought that specific biochemical properties of the misfolded prion protein characterise individual prion strains [3]. The ME7 and 22L prion strains have been previously characterized with respect to the patterns of neuronal loss induced in the hippocampus and cerebellum using light microscopy. The ME7 strain is known to induce extensive neuronal death in the CA1 region of the hippocampus. In contrast, the 22L strain has been reported to affect the cerebellum with loss of Purkinje cells. Both strains show clear microglial and synaptic changes in the hippocampus [4].

Synaptic pathology and degeneration in the hippocampus induced by the ME7 strain has been characterized at the electron microscopic level [2], [5]. We observed that the pre-synaptic compartment became disrupted with loss of integrity of the synaptic vesicles and then it was progressively enveloped by the post-synaptic density (PSD) of the dendritic spine [5]. Similar susceptibility of synaptic compartments also occurs in several other diseases associated with protein misfolding and aggregation [6]. It appears that the synapse is particularly vulnerable to the degenerative process highlighting it as a possible avenue for therapeutic intervention [7].

The susceptibility of distinct neuronal types has emerged as an important consideration in understanding these complex neurodegenerative diseases, which progress along defined anatomical routes [1], [4],[6]–[8]. The cerebellum has a well-defined synaptic circuitry and has been rigorously studied in developmental and post-developmental processes [9]. Understanding how a protein misfolding disease may impact on the cerebellum is important because of its involvement in perception, attention and other higher cognitive tasks [9], which might contribute to disease symptoms.

In Alzheimer’s disease (AD), reduced Purkinje cell density, atrophy of the molecular and granular cell layer [10] correlated with both clinical severity and duration of the disease, suggesting that the cerebellar changes are involved in disease progression [11]. Creutzfeldt-Jakob disease (CJD), the most common prion disease in humans, is typically associated with cerebellar alterations, including focal loss of Purkinje cells, spongiform degeneration in the molecular layer, moderate loss of granule cells and gliosis of astrocytes and Bergmann glia [12]. Studies with the Golgi method showed reduction of dendritic arbors, as well as hypertrophy and flattening of major dendrites of Purkinje cells [13].

Using the mouse-adapted 22L prion strain we have studied the progression of synaptic degeneration in the hippocampus and cerebellum. This approach allows us to address region-selective degenerative processes involving distinct neuronal types. Our findings show that in the pathogenesis of a single prion strain pre-synaptic compartments within the hippocampus are more susceptible to chronic neurodegenerative changes than other compartments, while in the cerebellum Purkinje cell dendrites are first to exhibit hallmarks of degeneration. Notably, this occurs without obvious changes in biochemical and ultrastructural properties of the major pre-synaptic elements such as observed previously in hippocampal CA1 neurons [14].

## Results

We have previously described the specific pathology of glutamatergic synapses induced by the ME7 strain in detail [5]. We next explored whether the steps of synaptic loss in the ME7 model represent a more general mechanism that could be reproduced using a different prion strain. The 22L prion strain targets the cerebellum [2], [4], therefore we aimed to characterize the synaptic pathology of the cerebellum and hippocampus at two time points, 12 (ES) and 18 (LS) weeks postinjection (p.i.).

### Degeneration in the Cerebellum of 22L-animals, Immunocytochemistry

The differential dendritic and synaptic degeneration in the cerebellum was first identified by immunolabeling of the Purkinje cells and the parallel fibres. Calcium-binding protein calbindin D-28k (calbindin) is expressed abundantly not only in Purkinje cells but also in climbing fibres [15], its selective genetic deletion from cerebellar Purkinje cells results in distinct cellular and behavioural alterations [16].

**Figure 1:**
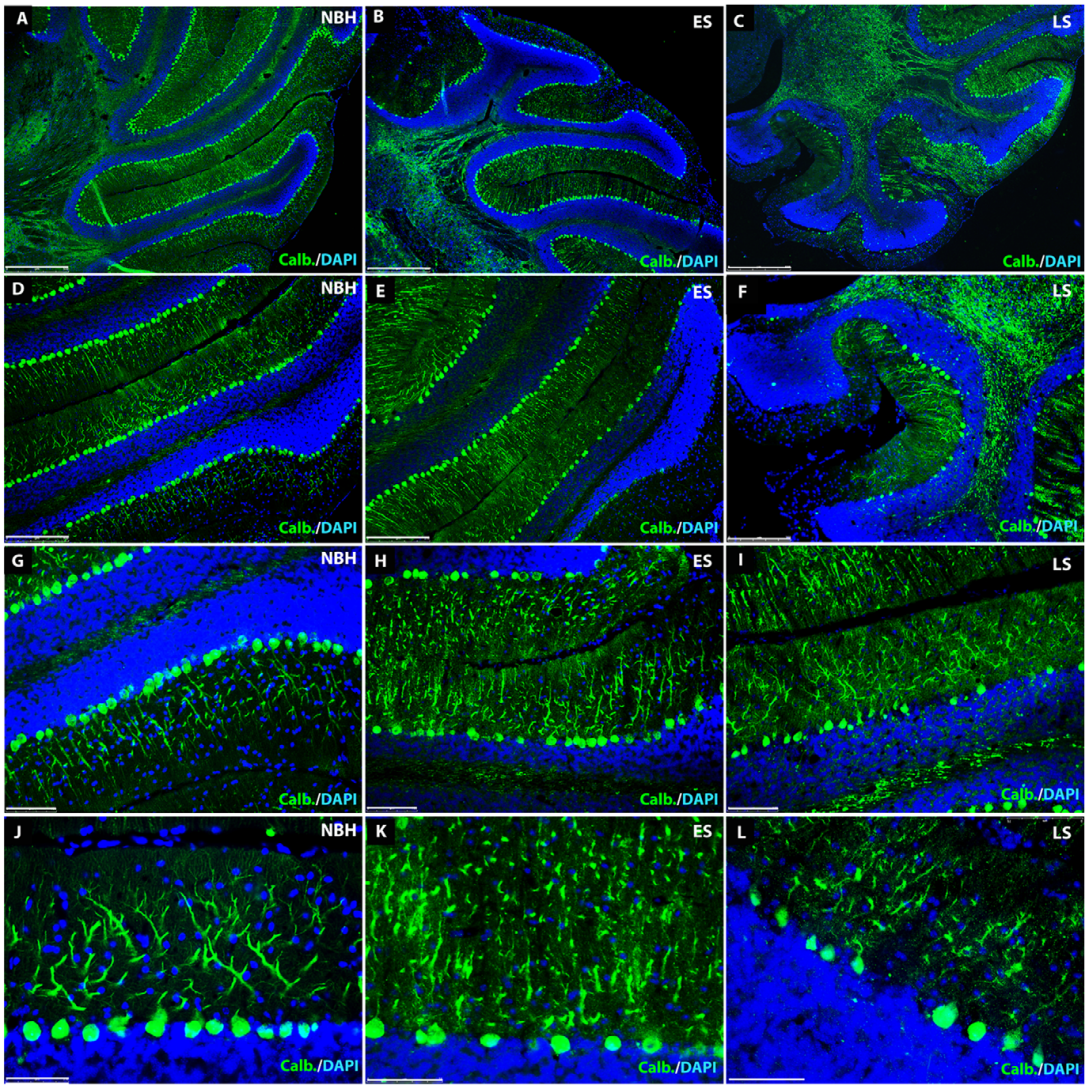
Cellular anatomy of the cerebellum in NBH- and 22L-animals. Calbindin-positive (green) Purkinje cells and nuclear stain (blue) of the cell-rich granule cell layer contrasts with the cell-sparse molecular layer illustrating the laminar cerebellar anatomy with the well defined arborizations of Purkinje cells penetrating deeply into the superficial molecular layer of a NBH control animal (18 weeks p.i.) at increasing magnification (A, D, G, J). Note the almost continuous layer of Purkinje cell bodies. Scale bars: 500 µm (A); 250 µm (D); 100 µm (G); 75 µm (J). In 22L-animals at ES (12 weeks) p.i. Purkinje cells and their dendrites appear relatively normal with calbindin immunoreactivity (green) relatively uniformly distributed within the cell (B, E, H). However, when observed with a 60× objective (K), fragmentation of the dendritic arborizations, revealed by a discontinuous labelling pattern, becomes apparent. Note that there are some gaps in the layer of the Purkinje cell bodies suggesting the early stages of Purkinje cell loss. Scale bars: 500 µm (B); 250 µm (E); 100 µm (H); 75 µm (K). At LS (18 weeks) p.i. in 22L-animals calbindin immunoreactivity (green) reveals a severe dendritic pathology in the molecular layer of the cerebellum (C, F, I, L). Some calbindin-positive arborizations appear to be disconnected from their neuronal somata (nuclear stain, blue) (F, I). The Purkinje cell layer, which in NBH-animals shows close, regularly spaced somata, reveals areas completely devoid of calbindin labelling, indicating a loss of neurons. The loss of the large DAPI-stained cell nuclei indicates that this is a loss of the Purkinje cell rather than a loss of the calbindin staining. Numerous calbindin-positive Purkinje cell somata appear to lack any primary dendritic process (L). Scale bars: 500 µm (C); 250 µm (F); 100 µm (I); 75 µm (L).

In control, normal brain homogenate-injected (NBH)-animals, at 18 weeks (LS) p.i. calbindin immunoreactivity was specifically present within cell bodies of Purkinje cells and continuously filled dendritic arborizations in the molecular layer (Figure 1A, D, G, J). In 22L-animals however, the distribution of the calbindin immunoreactivity at ES p.i. revealed changes in dendritic morphology and showed irregular labelling, which appeared as isolated segments rather than continuous dendritic branches (Figure 1B, E, H, K). At this stage, the density of Purkinje cells somata in the cell layer appeared unaltered when compared to controls. These changes observed at ES became more pronounced as disease progressed. At LS p.i. there was a complete destruction of Purkinje cell arborizations in some areas of the molecular layer and this now coincided with a marked loss of Purkinje cells (Figure 1C, F, I, L). Previous studies have documented a greater than 50% loss of Purkinje cells at LS [4]: concomitant with the described degeneration and loss of the Purkinje cells, the molecular layer appeared to shrink.

**Figure 2:**
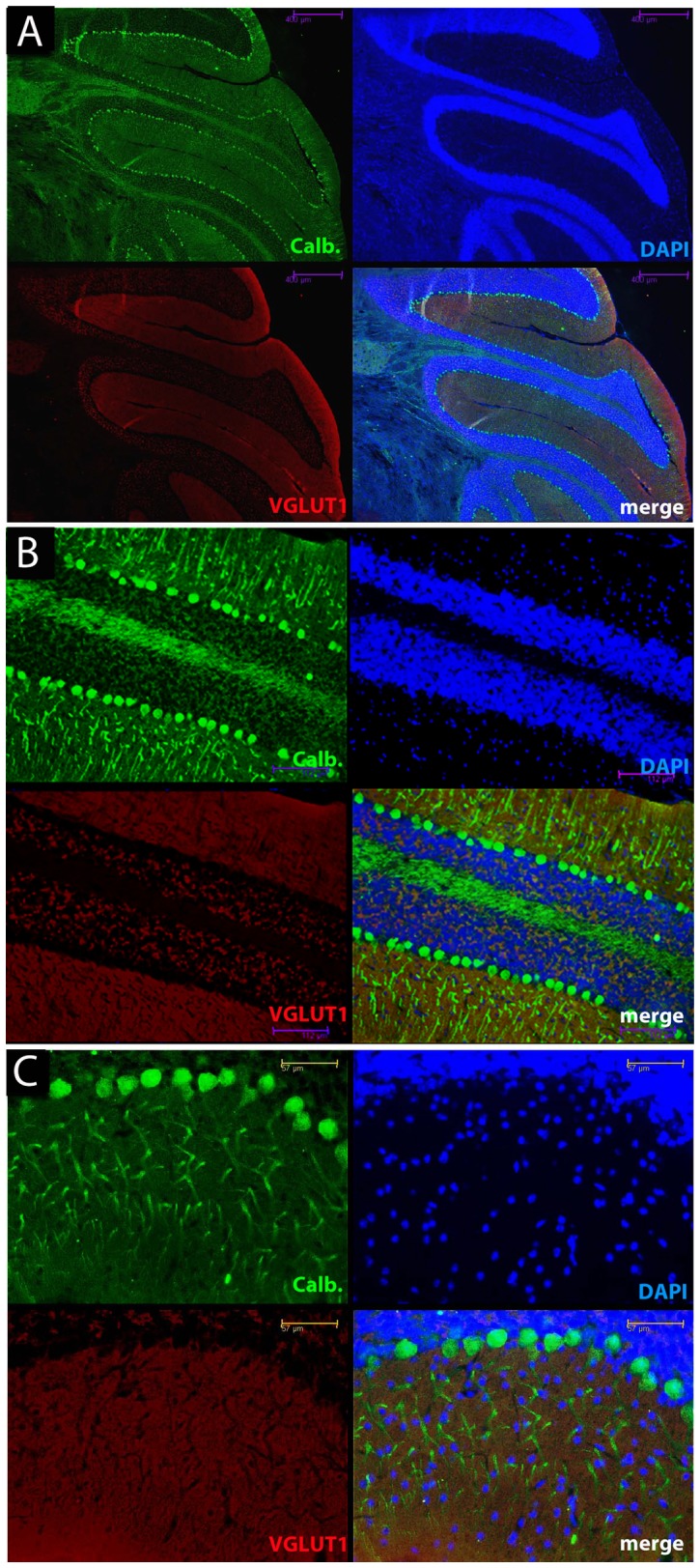
Immunofluorescence of cerebellar sections at increasing magnifications illustrates the relationship between calbindin-positive (green) Purkinje cell bodies, their dendrites and VGLUT1-positive (red) parallel fibres and synaptic terminals in the molecular layer, NBH-animals at 18 weeks p.i. The calbindin staining (green) reveals the Purkinje cells and the arborisation of their major processes in the molecular layer. The blue panel is DAPI staining of the cell bodies and the fourth panel shows the overlay of the three colours. VGLUT1 immunoreactivity (red) was abundantly present throughout the molecular layer and also in the granular layer associated with the cell somas of the granule cells (nuclear stain blue) that give rise to the parallel fibres. Scale bars: 400 µm (A); 112 µm (B); 57 µm (C).

**Figure 3:**
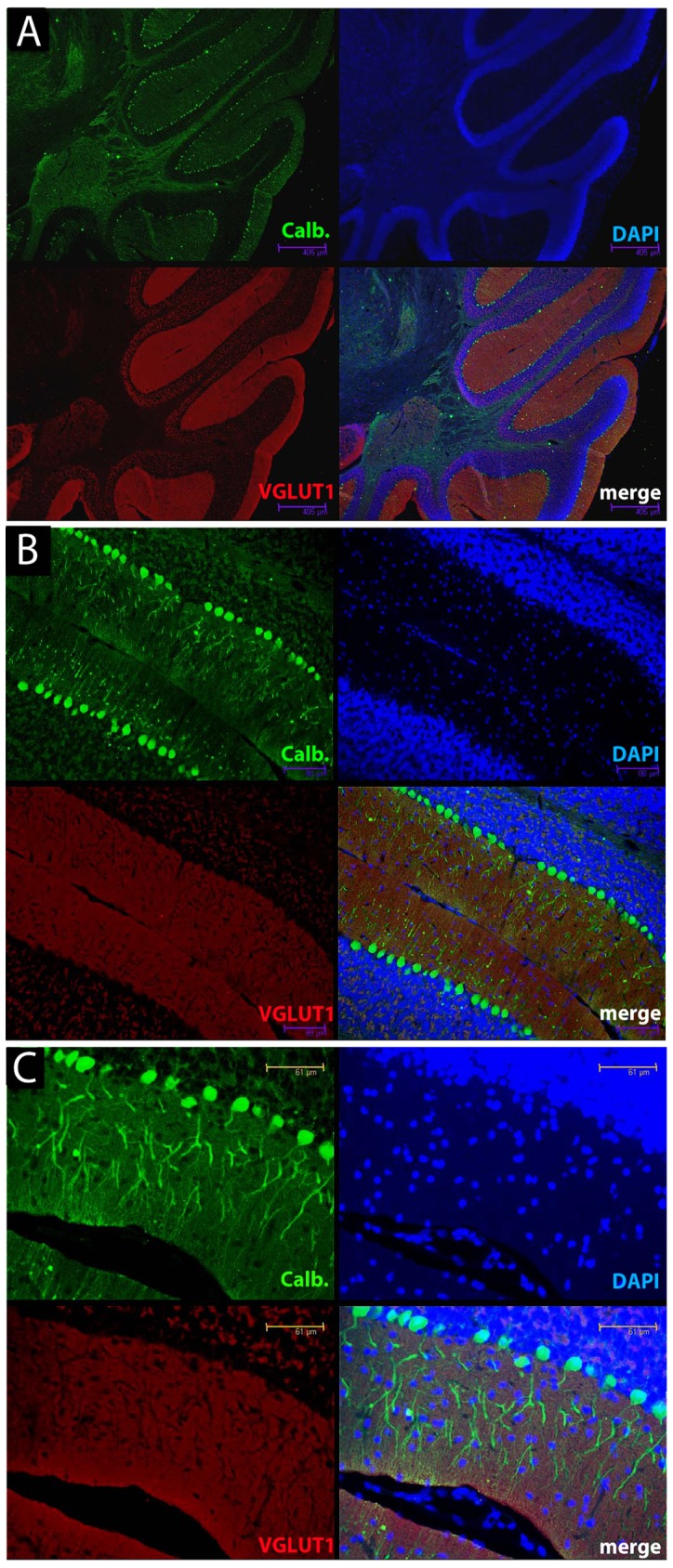
Calbindin and VGLUT1 staining in cerebellum of early stage 22L-animals highlights dominant dendritic pathology in early disease. Co-labelling of Purkinje cells using the anti-calbindin antibody (green) and parallel fibers (VGLUT1 antibody, red) at increasing magnifications from panel A to C in 22L-animals at ES p.i. The VGLUT1 immunoreactivity remains preserved in the areas of dendritic disintegration as revealed by discontinuous outlines of Purkinje dendrites (B, C). There is no significant loss of neurons at this time (nuclear stain, blue). Scale bars: 405 µm (A); 88 µm (B); 61 µm (C).

**Figure 4:**
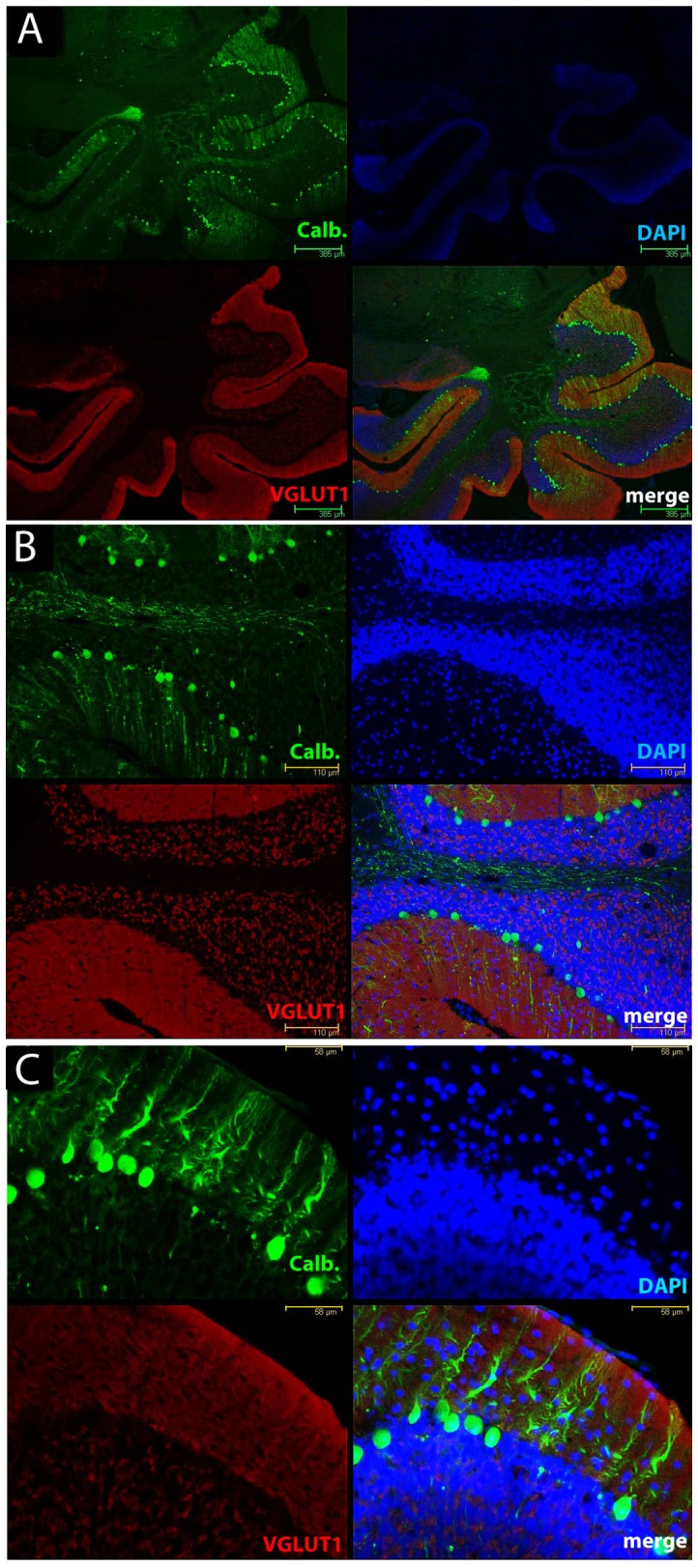
Calbindin staining in cerebellum of late stage 22L-animals highlights progressive dendritic pathology but limited presynaptic degeneration in VGLUT1 reactive parallel fibre synapses. Calbindin immunoreactivity (green) illustrates the severe loss of Purkinje cells dendrites in the molecular layer of the cerebellum and a reduction in Purkinje cell number (nuclear stain, blue) at LS p.i. in 22L-animals. VGLUT1-positive (red) parallel fibers and synaptic terminals are uniformly distributed throughout the molecular layer and do not exhibit any detectable alterations as revealed from images at increasing magnifications from A to C. Scale bars: 385 µM (A); 110 µm (B); 58 µm (C).

**Figure 5:**
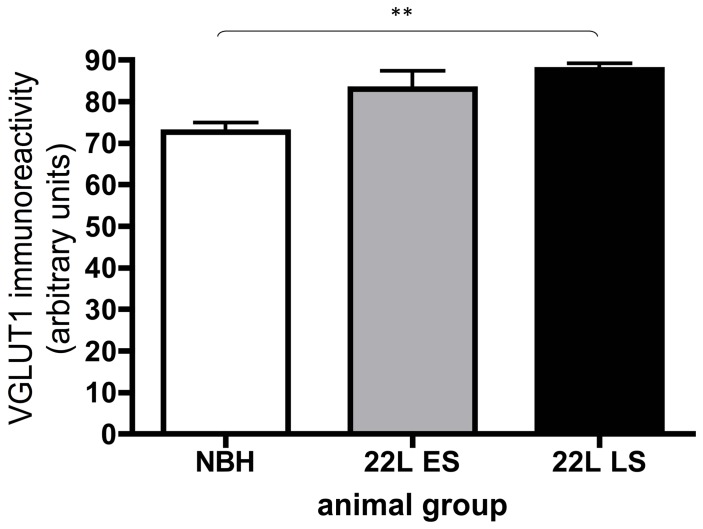
Quantification of VGLUT1 immunoreactivity in the molecular layer of the cerebellum from NBH, ES and LS 22L-animals. The analysis revealed no decrease in the quantified immunostaining consistent with observation that the presynaptic compartment is resistant to degeneration. In one-way analysis of variance with Tukey’s multiple comparison test (**P<0.01) a significant increase in the captured fluorescence was detected in 22L-animals at LS p.i. This increased staining is attributed to the pathology induced shrinkage of the molecular layer associated with disease progression.

We next explored the distribution of parallel fiber boutons that form the majority of excitatory synaptic connections in this layer. Different isoforms of a vesicular glutamate transporter can mediate glutamate uptake into the synaptic vesicles of excitatory neurons; however, parallel fibers in the molecular layer of the cerebellum have only VGLUT1 [17]. Using a specific antibody against VGLUT1 in sections from the cerebellum we investigated how expression changed during disease progression (Figure 2). In NBH-animals at 18 weeks p.i. calbindin immunoreactivity (green) revealed typical cerebellar anatomy, somata of Purkinje cells separating the cell-rich granule cell layer from the superficial molecular layer, almost devoid of any cells (nuclear stain in blue). VGLUT1 immunoreactivity (red) was abundantly present throughout the molecular layer and specifically labelled parallel fibers and their terminals on Purkinje cells (Figure 2A–C). Specific staining was also observed in the granule cell layer, associated with the cell bodies of the granule cells that give rise to the parallel fibres (Figure 2A–C). In 22L-animals at ES p.i, calbindin labelling (green) was altered as already described above. However, the pattern of VGLUT1 specific antibody staining (red) was unaltered relative to the staining in NBH-animals, even in the areas of Purkinje cell dendritic disintegration (Figure 3A–C). At LS p.i. the immunoreactivity of calbindin (green) revealed, as noted above, that the integrity of dendritic compartments was further compromised. Despite this observation, VGLUT1 staining (Figure 4A, B) was apparently unaffected in the regions where Purkinje cells and their arborizations were missing (Figure 4C). Densitometric measurement showed that the VGLUT1 immunoreactivity appeared to progressively increase and at LS p.i. this reached statistical significance (Figure 5; One-way analysis of variance with Tukey’s multiple comparison tests (N = 4/per experimental group). The apparent increase in VGLUT1 protein has to take into account the shrinkage of the molecular layer by about 20% relative to NBH-animals (compare Figure 2 and 4: data not shown).

### Prion Pathology in Hippocampus and Cerebellum of 22L–animals, Electron Microscopy

**Figure 6:**
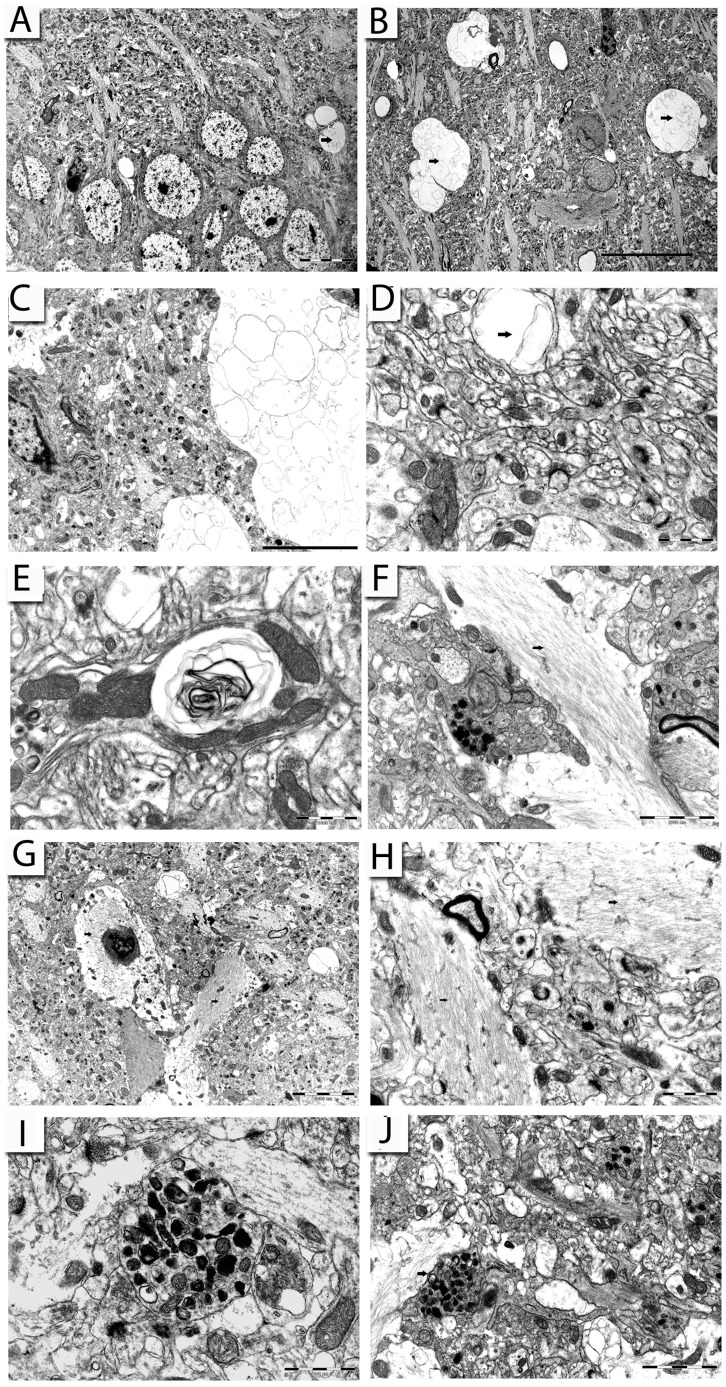
Electron micrographs of the stratum radiatum of the hippocampus and of the cerebellar molecular layer illustrating typical neuropathological hallmarks of prion disease. (A): A typical spongiform vacuole (arrow) within the pyramidal cell layer of CA1 region at ES p.i. (B): Numerous vacuoles containing whorled membrane fragments (arrows) within the stratum radiatum at LS p.i. (C): A large spongiform vacuole containing secondary vacuoles within the stratum radiatum at LS p.i. with ongoing synaptic pathology adjacent. (D): Proximal part of the molecular layer in the cerebellum at ES p.i. containing spongiform vacuole (arrow) with normal appearing synaptic structures in the vicinity. (E): Swollen neuronal terminal in the molecular layer of the cerebellum containing vacuole-like structure with numerous whorled membrane fragments in a 22L-animal at LS p.i. (F): Bergmann glia with bundles of fibrils (arrow) in the molecular layer of the cerebellum from a 22L-animal at ES p.i. (G, H): Numerous glial cells (arrows) within the stratum radiatum of the hippocampus with ongoing synaptic pathology at LS p.i. (I): A detail of a double-membrane enclosed autophagic vacuoles within the stratum radiatum of the hippocampus at LS p.i. (J): An autophagic vacuole (arrow) in the molecular layer of the cerebellum at LS p.i. in a 22L-animal. Scale bars: 10 µm (A, B); 5 µm (C, G); 1 µm (D, E, H, I); 2 µm (F, J).

Similar to the previous reports describing the ME7 strain [2], [5], the 22L-infected hippocampus contained typical hallmarks of prion disease pathology, although in contrast to the ME7 model there is little or no loss of neurons from the CA1 pyramidal cell layer [4]). Despite the absence of neuronal loss in the CA1 field, the hippocampal neuropil of 22L-animals contained spongiform vacuoles at early stages of the disease (Figure 6A), which were more abundant at LS p.i. (Figure 6B) and filled with many membrane fragments forming the disease-specific secondary vacuoles (Figure 6C). Within the cerebellum of 22L-animals, comparable numbers of these profiles were detected at ES p.i. (Figure 6D) and were also more abundant at later stages. Occasionally, vacuoles containing fragmented membranes were hard to distinguish from immature autophagic structures disintegrating within the Purkinje cell dendrites (Figure 6E). The progression of the pathology within the cerebellum was associated with activation of the Bergmann glia. With their cell bodies in the Purkinje cell layer and their processes filled with fine glial filaments (Figure 6F), they are similar to activated astrocytes observed in the hippocampus (Figure 6G, H). At late disease stage, the neuropil of both the hippocampus (Figure 6I) and the cerebellum (Figure 6J) had processes filled with numerous large darker organelles indicative of ongoing lysosomal degradation process.

### Synaptic Degeneration in the Hippocampus of 22L-animals

**Figure 7:**
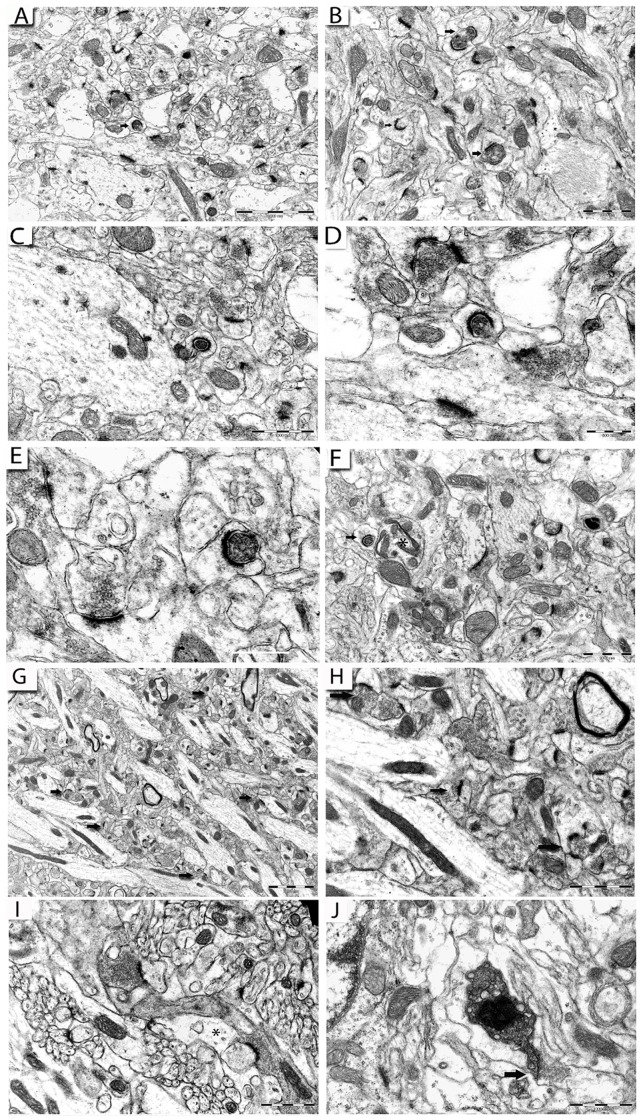
Pathology of synaptic terminals in the stratum radiatum and cerebellum of 22L-animals. (A–C): Electron micrographs illustrating numerous synaptic profiles in the stratum radiatum with inwardly curved post-synaptic membranes and electron-dense pre-synaptic terminals (arrows) at ES p.i. (D): A detail of the synaptic profile arrowed in A. (E): A degenerating synaptic profile with visible synaptic vesicles enclosed in a dark terminal at LS p.i. in stratum radiatum of a 22L-animal. (F): A synaptic profile (arrow) appears completely engulfed by highly curved PSD of a dendritic spine in the stratum radiatum at LS p.i., note a dystrophic neuronal terminal (asterisk) in proximity to the synaptic bouton. (G, H) Electron micrograph illustrating synapses in the stratum radiatum of NBH-animals at ES p.i., H: a detail of the panel G. Pre-synaptic terminals are filled with small round vesicles (<40 nm in diameter) and appose PSDs within an intact neuropil. (I, J): Type I synaptic junctions in the molecular layer of the cerebellum at ES p.i. (I) and at LS p.i. (J) in 22L-animals. Normal appearing pre-synaptic terminals without any hallmarks of pathological process filled with small vesicles appose structurally intact post-synaptic membranes on spiny branchlets of Purkinje cells; post-synaptic compartments of every bouton are closely enveloped by a process of one Bergmann glia cell that appears almost completely translucent (asterisks). J: A degenerating synaptic terminal filled with swollen vesicles and electron-dense cytoplasm (arrow). Scale bars: 2 µm (A, G); 1 µm (B, C, F, H–J); 0.5 µm (D); 0.2 µm (E).

In the electron microscope the synaptic alterations in the stratum radiatum of the 22L–infected hippocampus, were virtually identical to those that we have described in the hippocampus of animals injected with the ME7 prion strain [5]. As disease progressed from an ES (Figure 7A–D) to LS (Figure 7E, F), increasing numbers of dark, degenerating pre-synaptic terminals were associated with markedly curved PSDs, which in some instances completely surrounded the pre-synaptic element. The pre-synaptic elements no longer contained intact, resolvable pre-synaptic vesicles although the pre-synaptic membrane appeared to be intact and remain in a close apposition to the PSD. As previously described in the ME7-animals the cytoplasm of the dendritic spines enveloping the degenerating pre-synaptic terminals appeared normal and showed no signs of degeneration. The degenerative process selectively targeted Type I, mature, glutamatergic synapses with prominent PSD structures [18]. In control NBH-animals, Type I synapses within the stratum radiatum have characteristic, almost linear PSDs and small round vesicles (approximately 40 nm in diameter) in translucent pre-synaptic terminals (Figure 7G–H).

### Degeneration of Dendrites in the Cerebellum of 22L-animals

In the cerebellum we saw no evidence of dark, electron-dense pre-synaptic profiles or abnormally shaped post-synaptic PSDs (Figure 7I). The parallel fiber boutons are small and uniform in size with clustered vesicles in terminals; they synapse on the spiny branchlets with characteristic prominent, slightly outwardly curved (convex) PSDs, usually containing smooth endoplasmic reticulum. Among many hundreds of synaptic profiles inspected, only two electron-dense pre-synaptic terminals filled with pleiomorphic, swollen vesicles were observed (Figure 7J).

**Figure 8:**
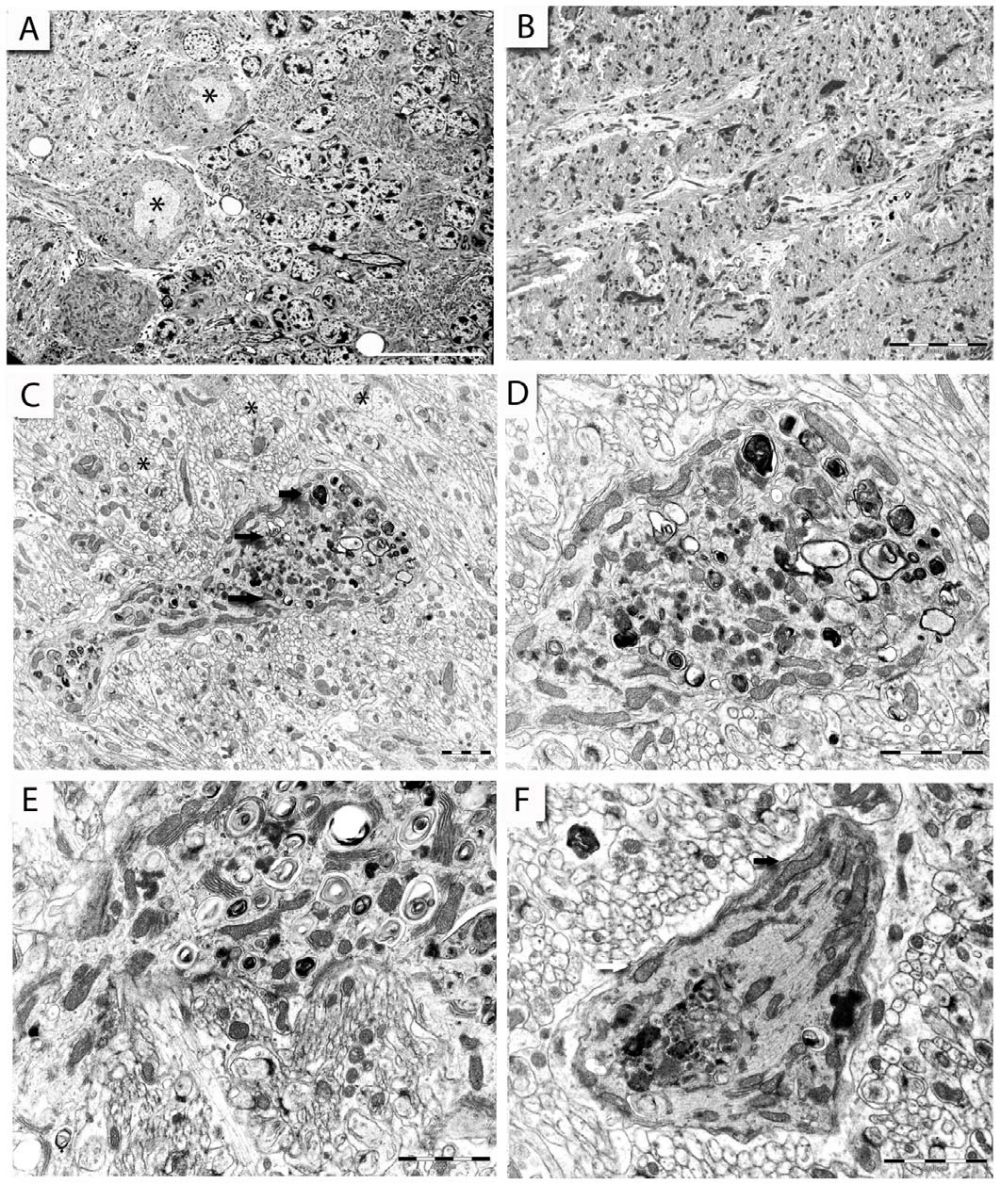
Dendritic pathology in the cerebellum of 22L-animals. Electron micrographs illustrating cerebellar ultrastructure (A, B). (A): Ultrastructure of the granule cell layer containing numerous granule cell bodies on the right and Purkinje cell layer with several cell bodies (asterisks) on the left side, NBH-animal 18 weeks p.i. (B): Molecular layer containing parallel and climbing fibers projections contacting Purkinje cells dendrites in 22L-animals at ES p.i. (C): A disintegrating dendritic segment of a Purkinje cell within the superficial molecular layer at ES p.i. filled with various lysosomal-like profiles (arrows), synapses in the proximity (asterisks) appear unaltered (a detail of the dendrite is shown in panel D). (E): A detail of disintegrating dendritic segment at LS p.i. in the molecular layer, note numerous electron-dense, dark profiles and vacuoles with whorled membrane fragments in the dendritic cytoplasm, general hallmarks of autophagic process. (F): A dystrophic Purkinje cell dendritic segment with mitochondria clustered against the plasma membrane (arrows), synaptic terminals in the proximity appear normal, molecular layer at LS p.i. Scale bars: 5 µm (A); 10 µm (B); 2 µm (C–F).

The cerebellum has a well-described organization, with clearly defined cell types, afferent and efferent pathways, and intrinsic cortical circuits. Axons of granule cells are unmyelinated, like the Schaffer collaterals innervating CA1 pyramidal neurons in the hippocampus; they ascend from the granular to the molecular layer, which is relatively sparsely populated with cells. In the molecular layer, they branch into parallel fibers that run perpendicular to the Purkinje cells arborization and contact their dendrites via dendritic spines (Figure 8A–B). Parallel fibers are glutamatergic and they usually excite a row of Purkinje cells [19]. In addition, each Purkinje cell is contacted by one climbing fiber, which exclusively originates from the contralateral inferior olivary nucleus and synapses directly on the proximal dendrite, unlike the parallel fiber they do not penetrate deeply into the molecular layer [20]. Numerous discrete synaptic junctions formed on stubby spines are characterized by a high density of vesicles widely distributed within a darker matrix and some isolated dense core vesicles, normally not observed in parallel fiber terminals [21].

It was immediately apparent that the dendritic segments in the superficial molecular layer (Figure 8C–F) of 22L-animals were abnormal and had conspicuous signs of degeneration at both ES and LS. There was a marked increase in the number of degenerating profiles in LS animals (18–19 weeks p.i.) that correlated with previously reported death of Purkinje cells [4]. The dendrites were conspicuously filled with double membrane-enclosed vacuoles and these vacuoles showed a strong resemblance to autophagic vacuoles, which are potentially digesting the dendritic segments from inside (Figure 8E).

In previous studies the ultrastructure of neuronal mitochondria appeared sensitive to disease progression. Mitochondria that are present in abundance and uniformly distributed in control Purkinje dendrites, were abnormally clustered under the plasma membrane in 22L-animals. This redistribution of mitochondria is indicative of an ongoing degenerative process apparent at ES (Figure 8F). This observation is consistent with findings in post-mortem samples from individuals with CJD [13].

Despite the significant pathology of the Purkinje cell dendrites, there were no identifiable concomitant ultrastructural alterations of the innervating parallel fibre.

The light microscopy and electron microscopy described above suggest that the degeneration of the post-synaptic Purkinje cells is initiated prior to, or even in the absence of, degeneration of the pre-synaptic terminals and contrasts with the early synaptic pathology in the hippocampus in the same animals.

## Discussion

In a number of chronic neurodegenerative diseases such as AD [22], amyotrophic lateral sclerosis [23] and prion disease [2], [5] it has been proposed that the earliest changes involve the loss of synapses although it is often not clearly stated whether this involves the pre- or post-synaptic component. With the recognition that the degeneration of neurons may involve specific compartments with distinct pathways and mechanisms [24] it is important to define which element of the synapse, the pre- or post-synaptic component, is undergoing degeneration at any particular time during the progression of disease. In the ME7 model of mouse prion disease it has been demonstrated that one of first steps of neurodegeneration in the hippocampus is the degeneration of the pre-synaptic component of glutamatergic synapses in the stratum radiatum [2], [5]. The degeneration is characterised by the loss of vesicle integrity, shrinkage and degeneration of the pre-synaptic terminal with subsequent envelopment of the dying terminal by the PSD of the post-synaptic dendritic spine. It was not known whether this form of degeneration is present in other prion models or in other brain regions.

Our present findings show that synaptic pathology in the stratum radiatum of the hippocampus of 22L-animals, exhibits all features that were previously observed in ME7-animals. We have detected numerous, degenerating pre-synaptic terminals of Type I synapses that were progressively engulfed by PSD membranes of post-synaptic dendritic spines as they curved around the degenerating pre-synaptic element. Despite ongoing pre-synaptic deterioration, there was little or no evidence of dendritic pathology of CA1 dendrites at early stage. In agreement with our previous findings in the ME7 model, activated glial cells were not directly involved in phagocytosis of the degenerating synapses [5].

The 22L prion strain has been reported to predominantly affect cerebellar function and thus motor coordination tasks [25]. The anatomy and neurotransmitter systems regulating neuronal function in the cerebellum and hippocampus share some similarities, including en–passant glutamatergic synapses onto a dendritic spine, although the post-synaptic Purkinje cell is GABA-ergic. We were intrigued to discover that in contrast to the stratum radiatum of the hippocampus and despite significant amounts of PrP^Sc^ present throughout the cerebellum [4], the first detectable degenerative changes were in the post-synaptic dendrites rather than in the pre-synaptic compartment. Even at late disease stage only rare degenerating pre-synaptic elements were found. The preservation of the pre-synaptic compartment was also apparent in the immunocytochemical detection and preservation of the VGLUT1 protein.

Several possibilities could explain why the glutamatergic synapses of the molecular layer in the cerebellum are relatively resistant to the degenerative process. A distinct feature of Type I synapses in the molecular layer is the degree of their glial ensheathment. Bergmann glia, unlike the astrocytes in the stratum radiatum, tightly enclose individual synaptic boutons in the molecular layer and express high levels of glutamate transporters that limit diffusion of glutamate during its release from synaptic terminals. It is also thought that Bergmann glia cells consist of independent compartments capable of autonomous interactions tuned to the particular state of synapses they ensheath [26]. In addition, functional deficiency in Bergmann glia cells induced by mutant ataxin-1 protein causes a vulnerability of Purkinje cells to glutamate toxicity [27]. In contrast to the apparently neuron-autonomous synaptic removal within the hippocampus [5], several other reports have documented a non-cell-autonomous-degeneration affecting Purkinje cells [28], [29].

Taken together, it appears that Bergmann glia cells exert a powerful control over the function of Purkinje cells in a disease situation. We suggest that Bergmann glia cells could provide a protection by creating relatively self-sufficient compartmentalized microdomains, tightly ensheathing synapses in the molecular layer. We did not observe any morphological evidence to suggest that the function of Bergmann glia cells was adversely affected during neurodegeneration, thereby compromising their protective capacity.

Autophagy is a regulated lysosomal degradation process responsible for the turnover of long-lived proteins or organelles; in mammals, it is required for normal neuronal function and conditional inactivation of autophagy-related genes in the central nervous system leads to neurodegeneration [29], [30]. Some studies suggest that enhanced autophagy could be neuroprotective [31], while autophagic structures are frequently observed in increased numbers in dysfunctional or degenerating axons and dendrites in several neurodegenerative conditions, including AD [32], [33] and CJD [34]. In the electron microscope, autophagy appears in the form of numerous aberrant membrane structures, typically as double-membrane vacuole-like profiles in the axonal dystrophic or dendritic swellings. In animals with specific ablation of an essential autophagy gene *Atg7* [30] such structures occur relatively early in the cerebellum followed by a cell-autonomous Purkinje cell death. In our study autophagic profiles were frequently observed in disintegrating dendrites and could underlie their demise, however the exact mechanisms need to be studied further. In addition, no parallel comparison between the cerebellar dendritic ultrastructure in ME7 and 22L prion strains is currently possible due to the lack of detailed studies of ME7-infected cerebellum. It also remains to be clarified whether the degeneration process of the pre-synaptic terminal and the post-synaptic dendrite are mediated by the same molecular pathways, although the rather different appearance in the electron microscope suggests that they may not be identical.

In summary our study demonstrates that within a brain undergoing a chronic neurodegenerative process the first stages of degeneration may involve distinct neuronal compartments, either pre- or post-synaptic elements. The molecular events that predispose a neuron to undergo a particular type of degeneration when exposed to a toxic misfolded protein, in this case prion protein, pose an interesting and important challenge.

## Materials and Methods

### Animals

C57BL/6J (Harlan) female mice aged 8–10 weeks were obtained from Harlan Laboratories (Bicester, UK) and were group-housed within the animal care facilities at the University Southampton as previously described [4].

### Ethics Statement

All animals were housed under specific pathogen-free conditions and experiments were performed according to the guidelines approved by the UK Home Office regulations. Every effort was made to minimize any suffering to the animals.

### Surgeries

All operations were performed under the UK Home Office licence, as previously described [4]. Briefly, surgery was performed when the mice were 11–12 weeks old. Mice were anaesthetized by intraperitoneal injection of Avertin (2,2,2-tribromoethanol solution) (20 ml/kg) and mounted in a stereotaxic frame (David Kopf Instruments, Tujunga, CA, USA). Injections of 1 µl of homogenate (10% w/v in sterile PBS) of either normal C57 mouse brain (NBH-animals) or of a 22L prion agent-infected brain (22L-animals) were made bilaterally into the dorsal hippocampus with a 10-µl Hamilton syringe. The suspension was slowly infused and the needle was left in place for 2 min before being slowly withdrawn. Mice were placed in a heated recovery chamber and when fully recovered rehoused in groups and checked daily.

### Tissue Preparation

NBH- and 22L-animals were terminally anaesthetized with sodium pentobarbital and killed by perfusion fixation for transmission electron microscopy (TEM) at 12 (early stage, ES; N = 4) and 18 (late stage, LS; N = 4) weeks post injection (p.i.), to study the early and later stages of synaptic loss. A slow cardiac perfusion (20–30 min) was performed with fixative containing 3.4% paraformaldehyde, 1.25% glutaraldehyde, 0.2% picric acid in 0.1 M sodium phosphate buffer (final pH 7.2–7.4) immediately after short (<1.5 min) perfusion with heparinised saline, to minimise synaptic and glial ultrastructural changes that could be caused by brain hypoxia [5]. After approximately 1 h, the brains were dissected and post-fixed in fresh fixative overnight at 4°C. 150 µm thick coronal sections were cut on a vibratome. The area of CA1 pyramidal layer and stratum radiatum of the hippocampus and the Purkinje cell layer, the granular cell layer and molecular layer of the cerebellum were dissected out from every animal. Microdissected areas were washed in 0.1 M sodium phosphate buffer and post-fixed at room temperature for 1 h in 1% osmium tetroxide. Tissue blocks were dehydrated at room temperature through graded ethanols from 30% to 100% for 10 min each, including 1% uranyl acetate in 70% ethanol for 40 min. Blocks were placed in acetonitrile for 10 min and overnight in a 50∶50 solution of acetonitrile:TAAB resin, subsequently infiltrated with fresh TAAB resin for 6 h and polymerised at 60°C for 20–24 h. TAAB blocks were hand trimmed, followed by glass trimming at room temperature. Semi-thin (0.5–1 µm) sections were stained (1% v/v Toluidine Blue in 1% w/v Borax) and used to guide further cutting of the specimen block into ultra-thin sections (60–70 nm). Ultra-thin sections were placed onto either thin bar mesh copper palladium grids or formvar -coated slot grids or stained in Reynolds lead stain for 5 min. The grids were gently immersed three times in distilled water and then left to dry. Grids were examined using a Hitachi H7000 TEM with a MegaView III digital camera (Soft Imaging System) and subsequently processed using Adobe Photoshop software (Adobe Systems Incorporated, San Jose, CA). The identities of the coded blocks were only revealed to the observer after the data analysis was complete.

### Immunohistology

Coronal sections passing though the dorsal hippocampus and the cerebellum (10 µm) were cut from formalin fixed paraffin wax-embedded brains at 12 (early stage, ES; N = 4 mice) and 18 (late stage, LS; N = 4) weeks post injection (p.i.). Following dewaxing and rehydration of the tissue, citrate buffer-microwaving and trypsin (0.5% trypsin solution and 1% calcium chloride in distilled water for 15 min at 37°C) antigen retrieval was performed; after blocking (5% bovine serum albumin (BSA) in phosphate buffered saline (PBS) for 30 min), sections were incubated in a humid chamber overnight at 4°C with one of the following primary antibodies: anti-calbindin –D-28K (1∶100; C9848, Sigma-Aldrich, USA) or anti-vesicular glutamate transporter 1 (VGLUT1) (1∶100; catalogue number: 135 303, Synaptic Systems, Germany) and subsequently with Alexa Flour 488 (anti-calbindin-D-28K) or 546 (anti-VGLUT1) conjugated secondary antibodies (1∶300; Molecular Probes, USA). All antibodies were diluted in 0.25% BSA/PBS and washed off using PBS/Tween. The sections were covered with Vector-shield mounting medium containing DAPI, coversliped and imaged. Control sections were covered with the appropriate blocking serum followed by the secondary antibody while other sections were covered with the primary antibody diluted as above without addition of secondary antibody.

### Data Analysis and Statistical Analysis

VGLUT1 immunoreactivity was also analyzed at ES and LS p.i. following challenge with 22L-brain homogenate and at 18 weeks with NBH. Pixel density values of 100 µm^2^ of the molecular layer have been obtained from at least three cerebellar sections per animal and normalized to the pixel density of the background for each section using ImageJ software (U.S. National Institutes of Health, http://rsb.info.nih.gov/ij/download.html). The identities of the coded blocks and tissue sections were only revealed to the observer after the data analysis was complete. One-way analysis of variance (ANOVA) with Tukey’s multiple comparison tests were performed to compare final data between NBH (N = 4) and 22L-animals (N = 4) at each of ES and LS using GraphPad Prism software (GraphPad Software, Inc., CA).
